# RWI-GEO-VOTE: Small-scale data of the 2017 German federal election

**DOI:** 10.1016/j.dib.2024.111044

**Published:** 2024-10-18

**Authors:** Melinda Fremerey, Lukas Hörnig, Sandra Schaffner

**Affiliations:** a IW – German Economic Institute, Mailbox 10 19 42, 50459 Köln, Germany; b RWI – Leibniz-Institut für Wirtschaftsforschung e.V., Mailbox 10 30 54, 45030 Essen, Germany; c Ruhr-Universität Bochum, Universitätsstraße 150, 44801 Bochum, Germany

**Keywords:** German politics, Parliament, Political parties, Granular data, Grid-cell data

## Abstract

The RWI-GEO-VOTE dataset was collected to understand how the refugee inflows affected the vote share of parties in the 2017 German federal election. The dataset provides granular insights into the 2017 German federal election at the 1 km x 1 km grid cell level. Compiled by the Research Data Center (FDZ) Ruhr at RWI, it covers 175,758 grid cells and provides data on valid votes for all parties that could be elected. The data collection process involved reassigning votes from the polling station to the grid cell level, excluding absentee ballots. The data generation process included intersecting populated grid cells with constituency (Wahlbezirk, smallest German unit) shapefiles, adjusting adult population shares, and linking grid cell/constituency combinations to municipalities. Election results were incorporated using electoral district geometries or polling station addresses, with unassigned grid cells filled in based on municipal-level data. This dataset is of interest to researchers who wish to analyze election results at a spatially granular level.

Specifications Table

**Table:** 

Subject	Political Science
Specific subject area	2017 German Federal Election Results at 1 km x 1 km Grid Cell Level
Data format	Processed
Type of data	Table
Data collection	We obtained the number of votes by party at the polling station level and a shape file of the constituencies (*Wahlkreise*) from the Federal Election Commissioner (*Bundeswahlleite*r). The cities of Berlin, Hamburg, and Stuttgart provided us shape files of their electoral districts. The addresses of the polling stations outside these cities were manually collected from various internet sites and personal communications with municipal administrators. Shape files of municipal boundaries were provided by the Federal Office for Cartography and Geodesy (*Bundesamt für Kartographie und Geodäsie*, BKG). Finally, we used demographic information at the 1 km x 1 km grid cell level from the RWI-GEO-GRID dataset.
Data source location	RWI – Leibniz-Institut für Wirtschaftsforschung Essen Germany
Data accessibility	Repository name: Research Data Center Ruhr at RWI Data identification number (DOI): https://doi.org/10.7807/vote:share:2017:v1 Direct URL to data: **For peer review only**: https://my.hidrive.com/share/q7m46eiaux Instructions for accessing these data: To request data access, please send an e-mail with your name, institution, and address to fdz@rwi-essen.de or fill out the request form at https://www.rwi-essen.de/en/research-advice/further/research-data-center-ruhr-fdz/data-access. Data access will be provided free of charge. **For peer review only:** The link cannot be shared publicly, because for administrative reasons, we have to count data users. Editors and reviewers may access the data directly under https://my.hidrive.com/share/q7m46eiaux
Related research article	Melinda Fremerey, Lukas Hörnig, & Sandra Schaffner (2024). Becoming neighbors with refugees and voting for the far-right? The impact of refugee inflows at the small-scale level. Labour Economics. Volume 86. 102,467. ISSN 0927–5371. https://doi.org/10.1016/j.labeco.2023.102467.

## 1 Value of the Data

• The data contain results of the 2017 German Federal Election at the 1 km x 1 km grid cell level. It is the first time that data has been made available on a smaller regional level than electoral districts (Wahlbezirke) in combination with the geographical location for Germany.
• The data are useful to analyze differences in voting patterns at a highly granular level. It serves as an essential resource for policymakers, opinion research institutes, and researchers to analyze voting behavior in neighborhoods. Further, the data may be of interest for statistical analysis of spatial patterns of voting behavior.
• Researchers can reuse this dataset to analyze voting behavior in the context of different factors that are heterogeneous on a regional level like weather, population composition, environmental factors, distance to the poll station etc. The grid cell level is particularly useful to complement with other data containing small-scale environmental differences (both ecological and socio-economic/demographic differences). Due to the applied INSPIRE regulation it can be combined with German census data and Europen geospatial data from the European Commission. Both databases host data that is on the grid cell level.
• In addition to the analysis of voting behavior, the data can also be used as additional or explanatory data in many other ways. With the ability to combine the data with many other datasets, it is possible to link it to outcomes such as education, policy decisions, infrastructure investment, mobility behavior, housing prices, and much more.
• Finally, the methodology used can be applied to similar data to infer small-scale regional voting patterns.

## 2 Background

The data were originally collected for the analysis of the impact of refugee inflows on far-right vote shares at the neighborhood level in Fremerey et al. [1]. In order to compile an election dataset at the small scale level, it was necessary to obtain results that were previously only accessible at higher spatial scales. The 1km x 1 km grid-cell level was chosen, as socio-demographic information - including the refugee inflow – was available at this level.

## 3 Data Description

This article describes the dataset RWI-GEO-VOTE which includes the results of the 2017 German federal parliamentary election at the 1km x 1km grid cell level (based on ETRS89-LAEA). It consists of 175,758 grid cells and covers the number of valid votes for 34 parties – that is all parties that were registered for the federal election of 2017. These data are organized in one csv file (rwi_geo_vote.csv). The file is accessible via a data request at the FDZ Ruhr [2].

The dataset contains a grid cell ID, called r1_id, which uniquely identifies observations. Because grid cells do not perfectly match constituency or electoral district boundaries, they may be assigned to more than one entity. In these cases, the entity IDs are collapsed with an "&" sign. The resulting constituency and electoral district IDs are reported in the wahlkreis and nearest_lokal variables, respectively. The variable “lokal” indicates what share of the grid cell is assigned to a polling station as opposed to being filled with municipality level data. The variable “valid_votes” is the total number of valid votes assigned to the grid. The following variables are the number of valid votes per party. A quick overview of all variables is given in Table 1.

**Table 1:** Description of the table structure.

Variable	Description
r1_id	Grid-Cell ID
wahlkreis	ID(s) of constituency – if multiple apply, the IDs are separated by an “&”
nearest_lokal	Composed ID(s) of the nearest polling station, taking the form “name._.constituency ID._.electoral district ID._.municipality ID”, or if no polling station was assigned “munic_municipality ID”. If multiple apply, the IDs are separated by an “&”.
lokal	Number ranging from 0 to 1 indicating the share of the grid assigned to a polling station.
valid_votes	Number of total valid votes assigned to the grid
**Valid Votes per Party**^1^	Number of valid votes for a specific party assigned the grid (34 variables)

1 spd, cdu, csu, gruene, fdp, dielinke, npd, bueso, bp, mlpd, diepartei, piraten, tierschutzpartei, oedp, volksabstimmung, afd, dierechte, freiewaehler, pdv, sgp, addemokraten, tierschutzallianz, b, bge, dib, dkp, dm, diegrauen, du, mg, menschlichewelt, diehumanisten, gesundheitsforschung, vpartei.

**Fig. 1:**
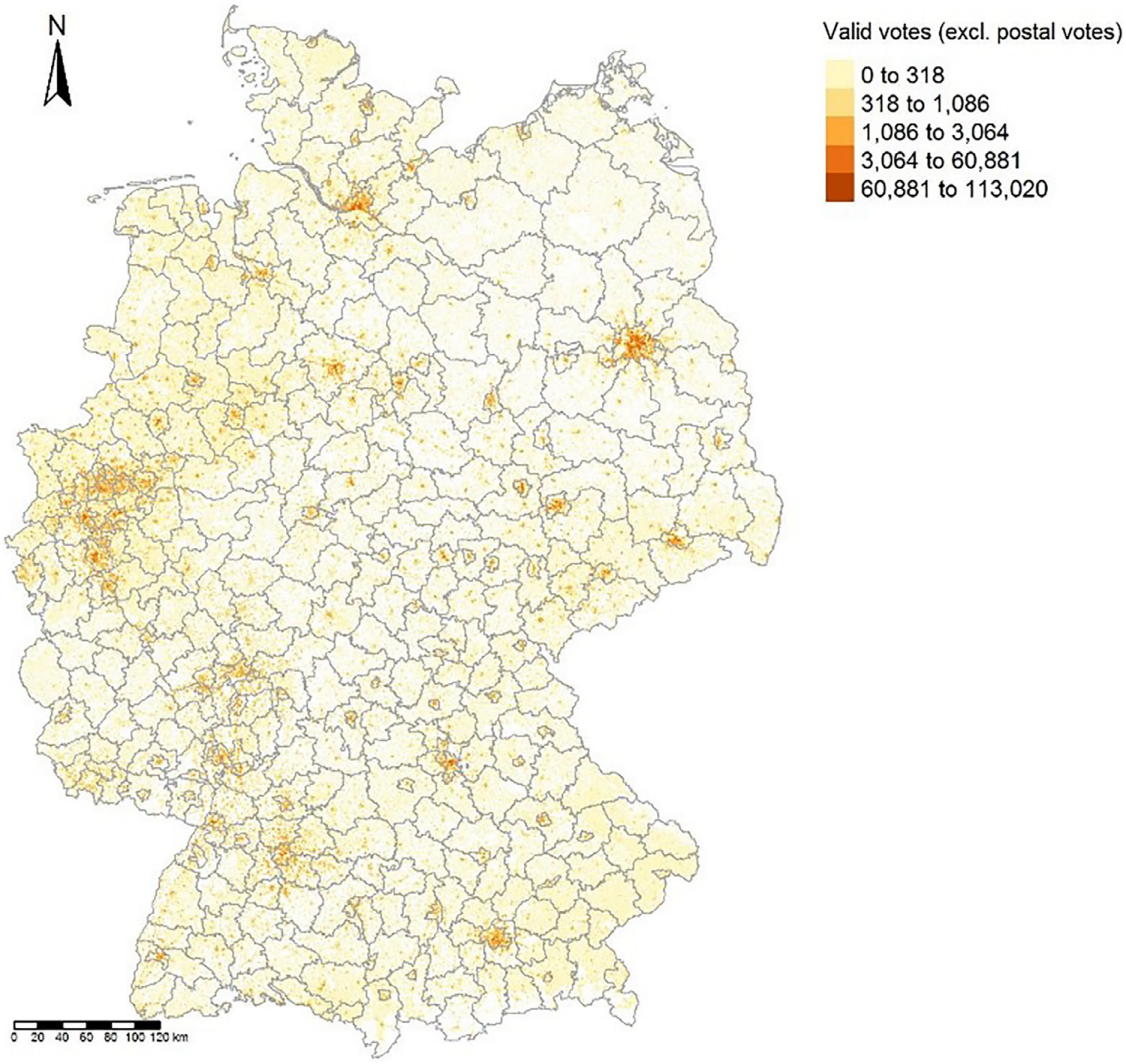
Map of number valid votes at grid cell level. Notes: Grey lines indicate county borders as of 2017. © GeoBasis-DE / BKG 2018.

Fig. 1 shows the regional distribution of valid votes, excluding postal votes, of the 2017 federal election at the 1km x 1 km grid cell level.

## 4 Experimental Design, Materials and Methods

The research data center (FDZ) Ruhr at the RWI provides a unique dataset on the 2017 German federal election at the 1 km x 1 km grid cell level, the RWI-GEO-VOTE. The dataset contains number of votes by party obtained from the Bundeswahlleiter at the polling station level, which we reassign to the grid cell level. We include only valid votes casted at the polling station. Absentee ballots are not included because they are aggregated at a higher spatial level and therefore, cannot be assigned at grid level. Differences between the RWI-GEO-VOTE and the official election remain negligible.

The generation of the dataset is as follows. We intersect populated grid cells with a shapefile of constituencies (Wahlkreise). Information on the population stems from the data set RWI-GEO-GRID [3]. We divide grid cells, which belong to more than one constituency, into the parts of the grid cell belonging to each constituency. For these grid cells, the adult population was adjusted by the share of the grid cell within one constituency, that is if a third of one grid cell is in constituency A and two thirds in constituency B, and nine adults live in the grid cell, then three adults are allocated to grid cell A and six to grid cell B. The implicit assumption is that population is equally distributed within the grid cell. While this assumption is simplifying, it is reasonable because of the small regional scale of the 1km x 1 km grid cell. Afterwards each grid cell-constituency combination is assigned to a municipality by its geographic center.

**Fig. 2:**
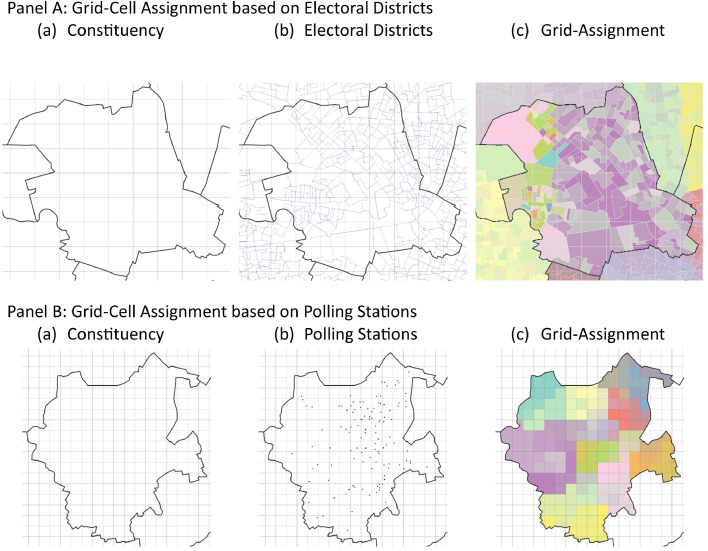
Illustration of assignment procedure of election results to grid cell-constituency combinations. Notes: Grey lines indicate constituency borders, blue lines borders of electoral districts and blue dots indicate the location of polling stations. The colors indicate the assigned electoral district to the grid cell(‘s share). © GeoBasis-DE / BKG 2018.

Election results are added to the grid cell-constituency combinations in two different ways depending on the type of the original source. If geometries of the electoral districts are available, the grid cell-constituency combinations of the corresponding municipalities are intersected with the electoral district geometries. The votes were divided among the intersection in proportion to the adult population. Finally, the combinations are aggregated on grid cell level. If geometries are not available, the addresses of the polling stations are used. To ensure that no electoral district is allocated to a completely wrong grid cell, the procedure is repeated for each constituency-municipality combination separately. If addresses of all polling stations within a constituency-municipality combination are known, the polling station is allocated to the grid cell with the minimum distance between the grid cell center and the polling station. This is reasonable as electoral districts are defined in such a way that they comprise a maximum of 2500 inhabitants and they should be easily reachable for as many eligible voters as possible to take part in the election. We show these two procedures in Fig. 2 for the Berlin-Mitte constituency (Panel A, based on the shape file of the electoral districts) and the Mönchengladbach constituency (Panel B, based on the location of the polling stations). The colors indicate the assigned electoral district to the grid cells share. Remaining grid cells, i.e. where we have no shape file of the electoral district or do not know the addresses of all polling stations in the constituency-municipality combination, cannot be allocated to electoral districts. These grid cells are filled with their population proportionate share of the valid votes at the municipality level.

## Limitations

There are two main limitations: First, there is an unknown level of measurement error due to the lack of electoral district geometries. While assigning grid-cell-constituency combinations to the nearest polling station within the same municipality helps to avoid misspecification between municipalities, some grids may still be misallocated. Since the correct assignments are unknown, it is not possible to quantify this error. The second limitation is the incomplete coverage of polling station addresses for many municipalities. In these cases, the grid cells are filled with the votes at the municipality level multiplied by the grid's share of the municipality's adult population. This procedure is applied to both the number of valid votes and the votes for specific parties, resulting in a constant party share in these municipalities. It is worth noting that only 30 % of the grids are based on polling station level information.

## Ethics Statement

The authors have read and followed the ethical requirements for publication in Data in Brief and confirm that the current work does not involve individual human subjects, animal experiments, or any data collected from social media platforms.

## CRediT Author Statement

**Lukas Hörnig**: Conceptualization, Methodology, Software, Validation, Data Curation, Writing - Original Draft, Visualization. **Sandra Schaffner:** Conceptualization, Validation, Writing - Review & Editing, Supervision, Project administration, Funding acquisition. **Melinda Fremerey**: Conceptualization, Validation, Writing - Review & Editing.

## Declaration of generative AI and AI-assisted technologies in the writing process

During the preparation of this work the authors used ChatGPT and deepl.com to improve readability and language in general. After using these tools, the authors reviewed and edited the content as needed and take full responsibility for the content of the publication.

## Data Availability

Homepage Research Data Center Ruhr at RWIRWI-GEO-VOTE: Small-Scale Data of the 2017 German Federal Election (Original data).
